# Edge Computing and Blockchain for Quick Fake News Detection in IoV

**DOI:** 10.3390/s20164360

**Published:** 2020-08-05

**Authors:** Yonggang Xiao, Yanbing Liu, Tun Li

**Affiliations:** School of Computer Science and Technology, Chongqing University of Posts and Telecommunications, Chongqing 400065, China; liuyb@cqupt.edu.cn (Y.L.); litun@cqupt.edu.cn (T.L.)

**Keywords:** fake news detection, edge computing, permissioned blockchain, Bayesian networks, Internet of vehicles

## Abstract

The dissemination of false messages in Internet of Vehicles (IoV) has a negative impact on road safety and traffic efficiency. Therefore, it is critical to quickly detect fake news considering news timeliness in IoV. We propose a network computing framework Quick Fake News Detection (QcFND) in this paper, which exploits the technologies from Software-Defined Networking (SDN), edge computing, blockchain, and Bayesian networks. QcFND consists of two tiers: edge and vehicles. The edge is composed of Software-Defined Road Side Units (SDRSUs), which is extended from traditional Road Side Units (RSUs) and hosts virtual machines such as SDN controllers and blockchain servers. The SDN controllers help to implement the load balancing on IoV. The blockchain servers accommodate the reports submitted by vehicles and calculate the probability of the presence of a traffic event, providing time-sensitive services to the passing vehicles. Specifically, we exploit Bayesian Network to infer whether to trust the received traffic reports. We test the performance of QcFND with three platforms, i.e., Veins, Hyperledger Fabric, and Netica. Extensive simulations and experiments show that QcFND achieves good performance compared with other solutions.

## 1. Introduction

The message exchanges in Vehicular Ad hoc Network (VANET) help drivers perceive the traffic conditions, adapt driving routes, and avoid potential road hazard scenarios [1]. It seems that the road safety and the traffic efficiency can be easily achieved in VANET. However, there may exist malicious drivers and fake news [2], which can make VANET fail to fulfill our expectation. For example, malicious vehicles would transmit false Decentralized Environmental Notification Messages (DENMs) [3] to their advantage. Therefore, it is important to detect the fake news or false messages in vehicular networks for the good of drivers and the healthy operation of VANET.

Many data-centric trust management mechanisms have been proposed to address the credibility of messages in VANET. According to some decision logics, the combined trust level to an event is derived from multiple pieces of evidence such as reports from vehicles. The common approaches include majority voting, weighted voting, and Bayesian inference. In [4], the number of positive messages about an event indicates the trust level, for example, five for the event. In [2], the credibility of messages is calculated via the weighted sum, considering objective and subjective trust metrics. The opinions on an event from different nodes are aggregated by means of weighted majority voting [5]. The trust level to an event is denoted by the posterior probability of the event after receiving multiple pieces of evidence [6].

Nevertheless, these mechanisms are designed without considering the essential characteristics of VANET: random topology, self-organization, and dynamic connections. For example, we cannot guarantee that messages reach their destinations timely and finally. This brings about two possible problems. First, trust models may take a long time to infer the credibility of messages, generating outdated results. Second, trust models may not collect enough evidence for the decision logic, leading to inaccurate results. In addition, these mechanisms do not take the prior probability and the duration of a traffic event into account.

The development of edge computing [7,8] and blockchain [9,10] provides us with an opportunity to deal with the aforementioned problems. Both technologies complement each other’s advantages. Edge computing can be taken as cloud computing that is executed at the network edge and near data sources (such as vehicles). The Edge Computing Nodes (ECNs) host various resources (such as network, computing, and storage) and they can provide edge smart services with the help of virtualization technologies [7]. If we extend the functionality of Road Side Units (RSUs), convert them into ECNs, and implement the decision logics on them, all RSUs can collaborate on fake news detection. Specifically, false messages can be quickly identified with the help of load balancing. Hence, the first problem is solved. Moreover, this solution does not involve the communication with the remote cloud server, so the response time is further improved [11,12].

The blockchain is actually a distributed database containing transaction history, which is shared among peer nodes and regarded as the evidence that cannot be tampered with or forged [13]. If we further extend the functionality of RSUs, turn them into blockchain nodes, and put the evidence (i.e., reports or messages from multiple vehicles) for an event in the blockchain, every RSU in the network then has a complete copy of the up-to-date evidence, from which the accurate evaluation of the event can be derived. Hence, the second problem is solved.

In this paper, we propose a network computing framework named Quick Fake News Detection (QcFND) for Internet of Vehicles (IoV), which is the extended version of traditional VANET and supports more communication technologies and computing models [14]. IoV gives RSUs the ability to provide cloud data service to vehicles, which is exactly what this paper discusses. In general, QcFND achieves the features as follows. First, it can deal with the traffic burst due to the load balancing. When an event (such as traffic jam or icy road) happens, the vehicles in the proximity report the event with outgoing messages to the nearest RSU simultaneously, possibly resulting in RSU overload. Second, it features low transaction time because of Proof-of-Authority (PoA) [15]. To ensure the freshness of the evidence, the submitted reports on an event need to be recorded on the blockchain quickly. Third, it establishes a Bayesian network to handle the dynamics of the event and the evidence. From one moment to another, the state of the event can change and the evidence may become outdated.

Specifically, the main contributions of this paper are fourfold.

- A network computing framework named QcFND is proposed to quickly detect fake news in IoV. QcFND is the first trust management framework that comprehensively utilizes the technologies from Software-Defined Networking (SDN), edge computing, blockchain, and Bayesian networks. The storage and processing of the reports on traffic events are deployed on the network edge instead of on the cloud.
- An architecture of Software-Defined Road Side Units (SDRSUs) is designed for QcFND. Additional hardware and software components are integrated into traditional RSUs to support server virtualization and Software-Defined Networking (SDN). Moreover, distributed load balancer monitors the load of each SDRSU and redirects incoming messages accordingly.
- QcFND uses the permissioned blockchain as its distributed database for storing and disseminating evidence on events. Moreover, it adopts PoA as the consensus mechanism since PoA is fast and energy efficient. The peer nodes and orderer nodes that constitute the blockchain are created via server virtualization.
- QcFND exploits Bayesian network as its decision logic. We establish a causal network representing the probabilistic relationships among involved variables for fake news detection. Once the reports are submitted at different moment by different drivers with different trust levels, the probability of the presence of an traffic event is computed.

The rest of this paper is structured as follows. Related works are introduced in Section 2. Section 3 presents the overview of the system. Section 4 describes how the load balancing is achieved through SDN. Section 5 explains how the evidence is stored and disseminated through blockchain. Section 6 demonstrates the decision logic behind the fake news detection. The simulation and experiments are given in Section 7. Finally, Section 8 concludes the paper and outlines future work.

## 2. Related Works

This section presents related works from several fields, which inspire the design of our trust management framework QcFND.

### 2.1. Edge Computing

An RSU cloud is established and taken as vehicular cloud for the computational and communication infrastructure providing services to vehicles [16]. The RSU cloud includes traditional RSUs and specialized RSUs, and the latter is the former with additional components for SDN. To perform real-time tasks, an architecture for edge computing nodes is designed [17]. The architecture allows an edge server to decide whether the requested task should be transferred to the cloud or to another server. To address the problem of high estimation error and high communication cost in traffic sensing system, a mobile edge computing based service architecture is proposed to conduct the analysis on the network edge [18]. A scenario of Software-Defined VANET with 5G cellular network is presented, and the controller and the vehicles take optimizing forwarding strategies to balance the latency and the cost [19]. The best decisions are achieved via building a two-stage Stackelberg game and analyzing the game equilibrium. To decrease the delay of the computation off-loading, a cloud-based Mobile Edge Computing (MEC) mechanism in vehicular networks is given and a game approach is proposed to find the optimal strategies [11]. In addition, the arrival and the execution of the tasks on an MEC server are modeled via queuing theory.

### 2.2. Delay Models

While the blockchain technology is applied to the scenarios of vehicle networking, the performance analysis model is proposed to evaluate the average delay considering the retransmission [20]. That is, the total time is obtained when a transmission process makes several attempts before the transmission is successful. Under the assumption of the instantaneous transmission within the communication range, the end-to-end packet propagation delay in VANET is related to the number of catch-up times between disconnected vehicles. The Cumulative Distribution Function (CDF) of the time for a message to travel along a fixed length of highway is derived in [21]. Assuming that the information propagation process consists of the catch-up process and the forwarding process, analytical models are developed to estimate the information propagation process in a vehicular network [22]. The models show that various traffic conditions have a significant impact on the information delay.

### 2.3. Blockchain

The blockchain technology has been researched since Bitcoin emerged [23]. All the direct historical interactions and indirect opinions about vehicles are recorded on the blockchain as persistent evidence to evaluate the trustworthiness [24]. An energy blockchain for secure charging in smart community enables electric vehicles to publicly audit and share transaction records [25]. The blockchain adopts a reputation-based Delegated Byzantine Fault Tolerance (DBFT) consensus algorithm, i.e., the nodes must receive enough amount of confirm messages to verify the validity of the received block. Three major challenges hinder data sharing and storage in VANET, i.e., centralization, high cost for maintenance, and security threat. Therefore, a consortium blockchain is constructed to address the challenges and it exploits techniques such as the digital signature, the Practical Byzantine Fault Tolerance (PBFT), and the smart contract [26]. As payment records in vehicle-to-grid networks are exploited to analyze user behaviors and make decisions for the power supply, a blockchain-based payment mechanism is presented to realize the trade-off between privacy protection and information sharing [27].

### 2.4. Data-Centric Trust Models

Dempster–Shafer evidence theory is exploited to collect evidence on the trustworthiness of data and nodes and combine reports from different nodes [28]. A data-centric trust model calculates data trustworthiness according to an empirical formula, which takes the trustworthiness of vehicles, the correlative trustworthiness of events and vehicles, the Proximity in geographic location, and the proximity in time as input [2]. A blockchain based reputation system is presented for data credibility assessment [29]. The receiver decides to believe the broadcast message based on the sender’s reputation, which is updated according to the ratings from the vehicles in vehicular networks. A trust management scheme is proposed for vehicles to evaluate the credibility of received event messages in either objective or subjective manner [5]. The former is related to the validity of beacon messages and the subsequent movement of the sender; the latter is connected with the entity reputations of the senders. The peers in the network decide whether to believe a message according to the collected verdicts on the message [30]. That is, when the majority of the vehicles trust the message, the message is regarded as trustworthy. The incorrect traffic information is misleading and may put drivers in danger. Hence, Proof-of-Event consensus mechanism is proposed, it determines the validation for an event through a set of warning rules [31]. To differentiate between genuine and dubious messages, the receiver can calculate the trust levels by counting the popularity of the warning messages [4]. Drivers trust the message only if a predefined threshold is reached. The aggregated credibility and corresponding ratings of received messages from neighboring vehicles can be validated using Bayesian inference [32]. However, these schemes are heuristic and subjective, not considering the probability of traffic events and the relationship among events, vehicles, and reports.

Table 1 compares the works mentioned above to our approach in terms of the method used, defense against Sybil attack, and basis for judgement.

Note that this paper exploits Bayesian network to reason out the probability of traffic events. According to the best of our knowledge, there is no data-centric trust model for VANET that is developed with this approach. In our opinion, it is promising to evaluate the credibility of traffic reports via this more systematic and objective method.

There are mainly two ways to defend against Sybil attack: based on digital signature and based on reputation of entities. The former assigns public-private key pairs for vehicles to sign each message and authenticate themselves. The latter derives the trustworthiness of vehicles based on the history of behavior. In Table 1, “Yes” means that defense against Sybil attack is fully supported, the corresponding schemes, including ours, adopt the combination of both ways. “Partly” means that the feature is supported to some extent since the corresponding schemes use only the reputation-based approach, which sometimes cannot correctly identify malicious nodes.

## 3. Overview of QcFND

This section introduces the target, the network computing framework, and the work flow of QcFND.

### 3.1. Target

QcFND aims to detect fake news in IoV correctly and in a timely fashion. A traffic event happens at one moment and it may end up at another moment. Nearby vehicles perceive and report the event one after another, generating time-series messages. The vehicles are with different trust levels, i.e., honest vehicles submit genuine messages but dishonest ones may submit fake news to their advantage. Inferring the probability of the presence of the event at the current moment is the task of this paper.

### 3.2. Background

This section briefly introduces the major technologies involved in the work, i.e., SDN, edge computing, blockchain, and Bayesian networks.

In traditional networks, each network device has its own control plane and forwarding plane. Each device is configured or programed individually. With SDN, the original control planes are pulled out of the network devices and placed in a centralized controller used to program flows for the entire network. Consequently, we can make quick adjustments across the entire network.

In cloud computing, the devices at the edge of the network generate a massive amount of data that needs to be stored and computed at cloud data centers. This consumes much network bandwidth and results in the response latency. With edge computing, the computation and storage services are deployed close to where the data is generated, i.e., the edge devices, to save bandwidth and reduce latency.

A blockchain is defined as an immutable distributed database for recording transactions. A bunch of transactions are packaged into a block, which also includes the cryptographic hash of the prior block. This iterative process results in linked blocks, hence the name blockchain. The consensus algorithms dictate how to append blocks to the chain and have a significant impact on transaction time.

Bayesian network is a probabilistic graphical model that includes a set of state variables and a set of directed edges between variables. It is denoted by a directed acyclic graph; the strength of directed edges is represented as conditional probabilities. Bayesian networks take events that occurred as evidence and estimate the certainties for events that are not directly observable.

### 3.3. Framework

**Figure 1:**
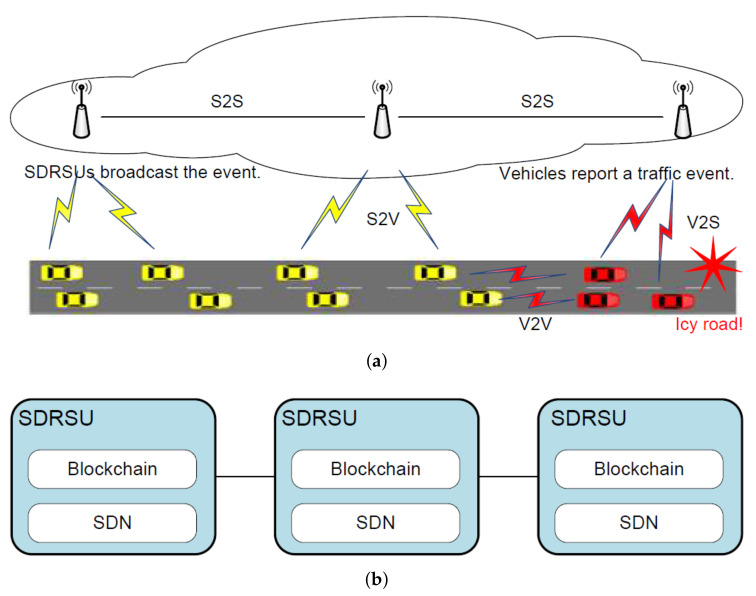
Quick Fake News Detection (QcFND) deployed in Internet of Vehicles (IoV). (**a**) A scenario where a traffic event happens. (**b**) Network computing framework.

The scenario is demonstrated in Figure 1a. There are two kinds of involved elements, i.e., SDRSU and vehicle. Among these elements, there are four types of connectivity, i.e., vehicle-to-SDRSU (V2S), SDRSU-to-vehicle (S2V), vehicle-to-vehicle (V2V), and SDRSU-to-SDRSU (S2S). Vehicles are responsible to report an event, and SDRSUs are responsible to collect these reports and find the truth from them. The SDRSUs are deployed on the road side and work together to provide services to the passing vehicles. Hence, QcFND can be taken as an instance of edge computing.

The proposed framework of QcFND is illustrated in Figure 1b. From a high-level point of view, SDRSUs communicate and collaborate with each other, and they constitute the main body of QcFND, a Software-Defined IoV (SDIoV). Each SDRSU contains two modules, i.e., SDN and blockchain.

The SDN module acts as an integral part in regulating the data flow in IoV and it implements the functionality of load balancing. It can acquire and analyze the information about the workload for all SDRSUs, and perform dynamic rerouting by sending corrective commands to SDN switch. On the contrary, it takes some while for the traditional network to converge before correctly rerouting the network traffic.

The blockchain module stores the evidence of events and calculates the possibility of the events. By putting the resources such as the storage and the computing at the network edge (SDRSU) and near the data source (vehicles), QcFND provides rapid responses for the traffic events, which are time-sensitive. On the contrary, the data has to travel back and forth between data sources and remote servers under the scenario of cloud computing.

### 3.4. Work Flow

**Figure 2:**
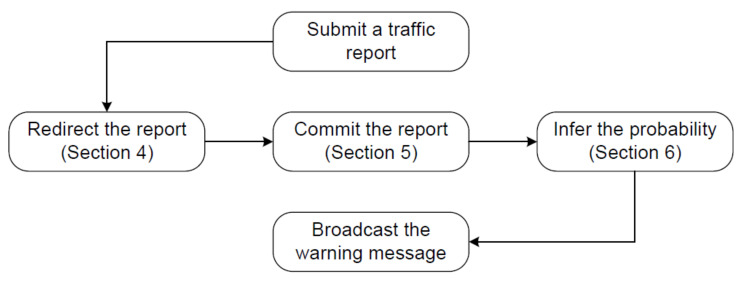
Work flow of QcFND.

Figure 2 demonstrates the work flow of QcFND and how its modules interact with each other. When a vehicle observes a traffic event, it submits a report about the traffic event to a nearby SDRSU. However, the nearby SDRSU may redirect the report to another SDRSU due to load balancing, not processing the report itself. The load balancer is implemented by SDN, and further details are given in Section 4. Once the report is delivered to blockchain nodes in an SDRSU, it will eventually be written to the blockchain on all SDRSUs. The transaction process is elaborated in Section 5. Whenever a new block is committed, the smart contract infers the possibility of the presence of the traffic event based on the algorithm of Bayesian network. The calculation process is presented in Section 6. Finally, SDRSUs broadcast the warning message on the event occurrence possibility.

## 4. Software-Defined RSU

In this section, we present the detailed architecture of SDRSU, on which edge computing and blockchain are implemented. The data structure of extended MAC table and the communication protocol among load balancer, SDN controller, and SDN switch are also introduced. Finally, we analyze the response time of QcFND using queuing theory and demonstrate the delay model.

### 4.1. Architecture of SDRSU

**Figure 3:**
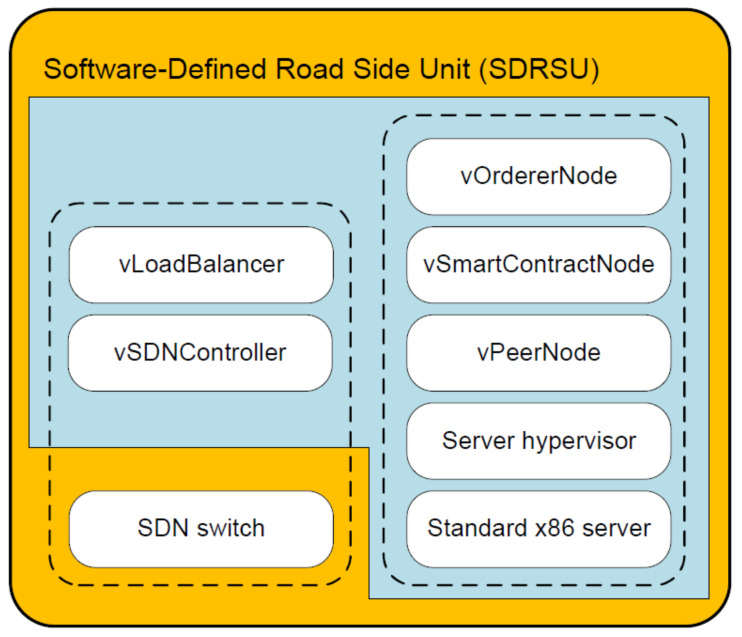
The architecture of Software-Defined Road Side Unit (SDRSU).

The components of SDRSU are shown in Figure 3. The SDRSU hardware consists of SDN switch and standard x86 server. The former represents the infrastructure layer of a network device in SDN and focuses on the data forwarding functionality. The latter represents the hardware layer during the server virtualization and is a collection of hardware resources for computing, storage, and networking. The SDRSU software is packaged as five virtual machines (VMs), including vSDNController, vLoadBalancer, vPeerNode, vSmartContractNode, and vOrdererNode. The server hypervisor is the virtualization layer sitting on the physical server and abstracts hardware resources into these VMs.

From the function view, SDRSU comprises two modules SDN and blockchain, denoted as two dotted box in Figure 3. The two modules are also illustrated in Figure 1b. In SDN module, SDN switch is decoupled from related software counterparts, vSDNController and vLoadBalancer, which represent the control layer and the application layer of network devices respectively. The vSDNController implements the centralized control of the network rather than having each network device make its own decisions [16]. The vLoadBalancer gathers the statistical information on SDN switch, and evenly distributes the workload throughout the network via the vSDNController.

After being redirected to an SDRSU, the traffic reports are processed by blockchain nodes. In the blockchain module, blockchain is implemented via three VMs, i.e., vPeerNode, vSmartContractNode, and vOrdererNode. The further explanation about them is given in Section 5.

### 4.2. Protocol

The protocol dictates the communication procedure among SDN switch, vSDNController, and vLoadBalancer. It specifies how to implement SDN network and how to achieve load balancing in IoV.

#### 4.2.1. Extended MAC Table and Messages

We use the extended MAC table to record the state information of SDRSUs. It helps us determine which SDRSU incoming reports should be best forwarded to. We assume that there is a waiting queue in SDN switch, which accommodates the reports that cannot be processed immediately [33]. When SDRSU gets more reports than it can handle, some reports have to enter into the waiting queue. We can choose the size of the waiting queue as an indicator of workload.

The extended MAC table is located in vSDNController and contains a set of state entries where each entry contains three fields, i.e., MAC address, ingress port, and queue size. The first field indicates the MAC address of vPeerNode, the second field denotes the port via which the vPeerNode can be reached, and the third field is the size of the waiting queue of SDN switch located in the same SDRSU with the vPeerNode. An example of state table is illustrated in Table 2.

The messages of the communication protocol are listed in Table 3. They are used during the extended MAC table preparation and the workload distribution, which are explained in Section 4.2.2 and Section 4.2.3 respectively.

#### 4.2.2. Update the Extended MAC Table

**Figure 4:**
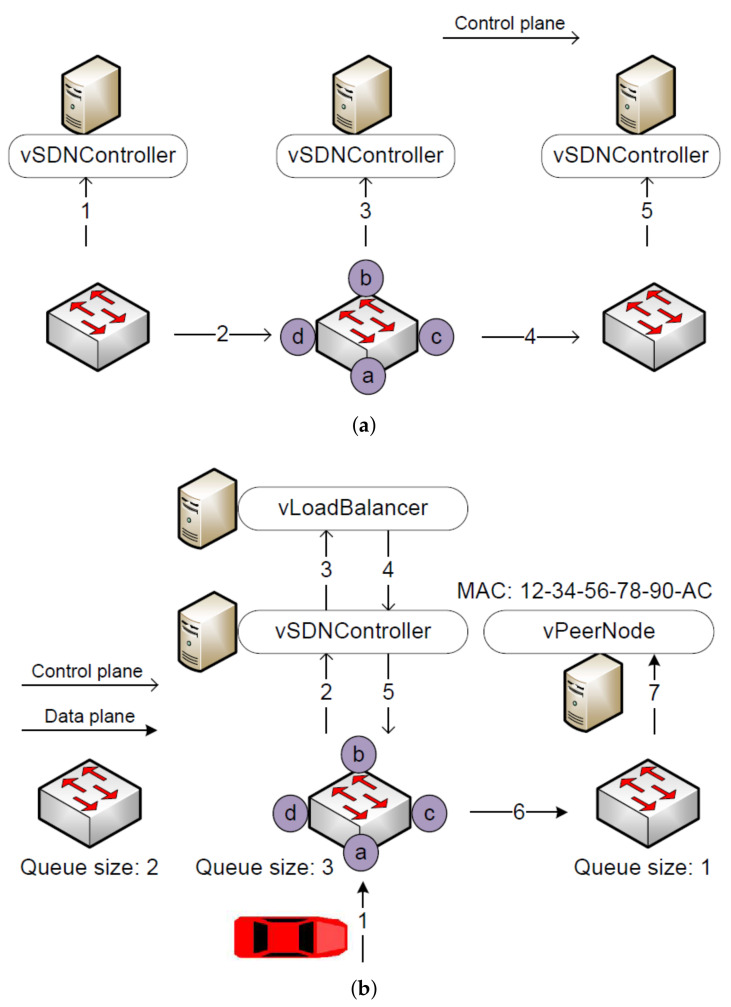
Communication protocol. (**a**) How to update the extended MAC Table. (**b**) How to distribute the workload.

The extended MAC table in vSDNController reflects the dynamic state of SDN switches. When the workload on a switch changes, the controllers in all SDRSUs have to be notified. In Figure 4a, the size of the waiting queue in the left switch changes, and it broadcasts the *SDNP_Queue_Size* message to every controller. All arrows represent *SDNP_Queue_Size* message, illustrating the propagation of the message on the control plane. While the arrows labeled 1, 3, and 5 indicate the internal communication in the SDRSU, those labeled 2 and 4 represent the communication between different SDRSUs. After each vSDNController receives this message, it updates its extended MAC table.

#### 4.2.3. Distribute the Workload

When an SDN switch receives a report, the local vSDNController and vLoadBalancer decide how to handle the message. It is forwarded either to the local blockchain nodes within the same SDRSU or to the remote blockchain nodes within another SDRSU. On average, the workload is evenly distributed among SDRSUs in the network.

In Figure 4b, we denote the control plane and the date plane with two types of arrows. The process of workload distribution is explained as follows. First, the red vehicle submits the report of a traffic event to the nearby SDRSU. Second, the local SDN controller is informed about the arrival of the report via the *SDNP_Packet_In* message. Third, the load balancer is asked which SDRSU the report needs to be forwarded to with *SDNP_Balance_In*, taking the load balancing into consideration. Fourth, the load balancer queries the extended MAC table and tells the SDN controller to redirect the report to the targeted port via *SDNP_Balance_Out*. Fifth, the SDN controller tells the SDN switch to forward the report to the targeted port with *SDNP_Packet_Out*. Sixth, the report is forwarded from the local SDRSU to the targeted SDRSU. Seventh, the SDN switch in the targeted SDRSU directly forwards the report to the local blockchain nodes. Note that we explain how the report is further processed in Section 5 and Section 6.

### 4.3. Delay Model

Since one of the main goals for QcFND is to quickly detect fake news in IoV, we present the delay model and compare the performance between the traditional network and our SDN network. In this paper, the total delay refers to the time between when a report on a traffic event is submitted by a vehicle and when the message on the event occurrence possibility is broadcast to all vehicles.

We make three assumptions for the simplicity. First, all types of connectivity have the same network latency $T_{n}$, and there is one connection per type of connectivity in the both networks. Second, all reports are redirected to another SDRSU in the SDN network, but they are forwarded to the cloud server in the traditional network. Third, the network delay in the same SDRSU is negligible.

In the traditional network, there are four types of connectivity, i.e., vehicle-to-RSU, RSU-to-cloud, cloud-to-RSU, and RSU-to-vehicle. Therefore, the total delay $T^{tra}$ is given by

(1) $$T^{tra}=4T_{n}+T_{r}^{tra}$$

where $T_{n}$ is the network delay on a connection and $T_{r}^{tra}$ is the response time for a report to be processed by blockchain nodes in the traditional network. In the SDN network, there are three types of connectivity involved, i.e., V2S, S2S, and S2V. Therefore, the total delay $T^{sdn}$ is given by

(2) $$T^{sdn}=3T_{n}+T_{r}^{sdn}$$

where $T_{r}^{sdn}$ is the response time in the SDN network.

The response time $T_{r}^{tra}$ and $T_{r}^{sdn}$ are different since the SDN network evenly distributes the workload throughout the network. Intuitively, a report does not have to wait for a long time before being processed in the SDN network. In the following, we utilize queuing theory to derive $T_{r}^{tra}$ and $T_{r}^{sdn}$.

**Figure 5:**
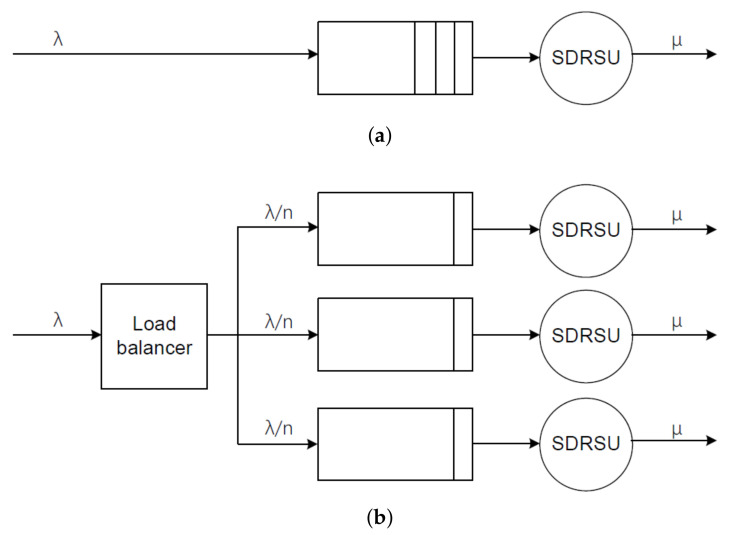
Delay model based on queuing theory. (**a**) Traditional network. (**b**) Software-Defined Networking (SDN) network.

In Figure 5a, reports are processed by an SDRSU in the traditional network, which is abstracted as an M/M/1/K queue. The interarrival times between two messages are assumed to be independent of each other and drawn from the exponential distribution with the parameter λ. The service times for each report are assumed to be independent of each other and exponentially distributed with the parameter μ. There is a single server and the system can only accommodate *K* reports. The reports are served in First in, First out (FIFO). If a new report arrives when there are already *K* messages in the SDRSU, the new report is dropped. According to [34], when $\rho =\frac{\lambda }{\mu }\neq 1$, $T_{r}^{tra}$ is obtained via

(3) $$T_{r}^{tra}=\frac{1}{\lambda }\frac{\rho }{1-\rho }\frac{1-\rho^{K+1}}{1-\rho^{K}}-\frac{1}{\lambda }\frac{\left(K+1\right)\rho^{K+1}}{1-\rho^{K}}$$

where $T_{r}^{tra}$ represents the mean time a report takes in the system, including waiting time and service time. Furthermore, the partial derivative of $T_{r}^{tra}$ with respect to λ is given by

(4) $$\frac{\partial T_{r}^{tra}}{\partial \lambda }\;=\;\frac{1}{{\left(\lambda -\mu \right)}^{2}}\;+\;\frac{K{\left(1-\rho \right)}^{2}\rho^{K-1}}{{\left[\left(\lambda -\mu \right)\left(1-\rho^{K}\right)\right]}^{2}}\;+\;\frac{K\left(K+1\right)\rho^{-(K+1)}}{{\left[\mu \left(1-\rho^{-K}\right)\right]}^{2}}$$

It can be easily seen that $\frac{\partial T_{r}^{tra}}{\partial \lambda }>0$ for λ>0 and μ>0.

When ρ=1, $T_{r}^{tra}$ is obtained via

(5) $$T_{r}^{tra}=\frac{K+1}{2\lambda }$$

which is the exact limit of the right side of Equation (3) at ρ=1, as proved via

(6) $$\lim_{\rho \to 1}\left(\frac{1}{\lambda }\frac{\rho }{1-\rho }\frac{1-\rho^{K+1}}{1-\rho^{K}}-\frac{1}{\lambda }\frac{\left(K+1\right)\rho^{K+1}}{1-\rho^{K}}\right)=\frac{K+1}{2\lambda }$$

According to Equations (3)–(6), we conclude that the response time $T_{r}^{tra}$ strictly monotonically increases with the increase of the arrival rate λ. This conclusion helps us analyze the performance of our SDN network.

In Figure 5b, the messages are evenly distributed among *n* SDRSUs in the SDN network, which is abstracted as *n* M/M/1/K queues. Each queue has exponential interarrival time distribution with the parameter $\frac{\lambda }{n}$ and exponential service time distribution with the parameter μ. For n>1, obviously we have $\lambda >\frac{\lambda }{n}$. According to the above conclusion about the monotonicity, the comparison of the response times is denoted as

(7) $$T_{r}^{tra}>T_{r}^{sdn}$$

Considering Equations (1) and (2), we eventually obtain

(8) $$T^{tra}>T^{sdn}$$

which indicates the improvement in the total delay after the adoption of our framework.

## 5. Blockchain

In Section 3, we mention that there are three blockchain nodes running on the same physical server. In this section, we explain how these VMs work together to implement the blockchain facility and provide fast data storage and computing services. Note that we utilize the technologies from Hyperledger Fabric [35] to build the blockchain and implement the consensus mechanism PoA.

### 5.1. Data Structures

**Figure 6:**
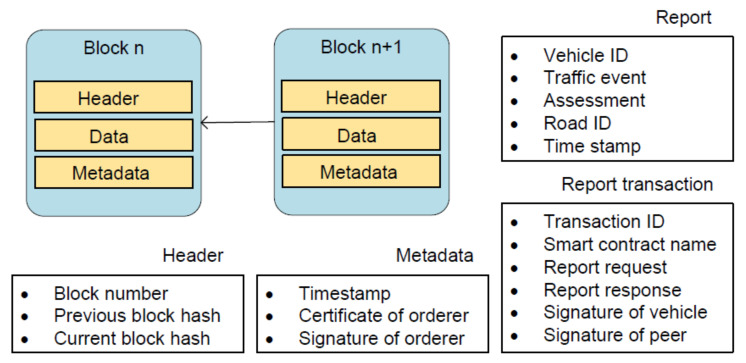
Data structures involved in blockchain.

The reports are submitted as the evidence on traffic events. When they are stored and processed on the blockchain, there are several data structures involved, such as reports, transactions and blocks, which are shown in Figure 6.

Detailed information about traffic events is given in a report, most of whose fields are self-explanatory. The assessment field has two possible values: positive and negative, denoting the occurrence and nonoccurrence of events from the view of drivers. Every report is included in a report request, which is sent to any vPeerNode for endorsement. The vPeerNode then generates a report response containing the execution results from vSmartContractNode, such as returned value, read set, and write set.

A fully endorsed transaction consists of six parts: transaction ID, smart contract name, report request, report response, and signatures of a vehicle and a vPeerNode. A block is composed of three parts: header, data, and metadata. The header includes block number, previous block hash, and current block hash. The data consists of a list of report transactions, which are invoked by vehicles. Timestamp as well as certificate and signature of a vOrdererNode constitute metadata.

The blocks are interlinked in a way that the previous hash of block n+1 is equal to the current hash of block *n*. The big feature of our blockchain is that blocks do not include a nonce field to satisfy the requirement of proof of work (PoW) [36]. By contrast, the validity of the blocks is guaranteed through the certificate and the signature field since PoA is adopted as the consensus protocol.

### 5.2. Transaction Processing

PoA dictates the whole process during which the submitted reports are eventually distributed across the network. That is, only authorized participants, i.e., vehicles and blockchain nodes, are eligible to take part in the process. These clients and servers are identified via certificates, which are used in the lifecycle of transactions.

The report request includes the certificate of a vehicle; the report response contains the certificate of an endorsing vPeerNode. When enforcing the access control, vSmartContractNode extracts the certificate from the report request, acquires the identity of the vehicle, and queries whether the access is allowed. As committing peers, vPeerNodes check the identity of who executes the report request and the identity of who assembles report transactions into a block.

**Figure 7:**
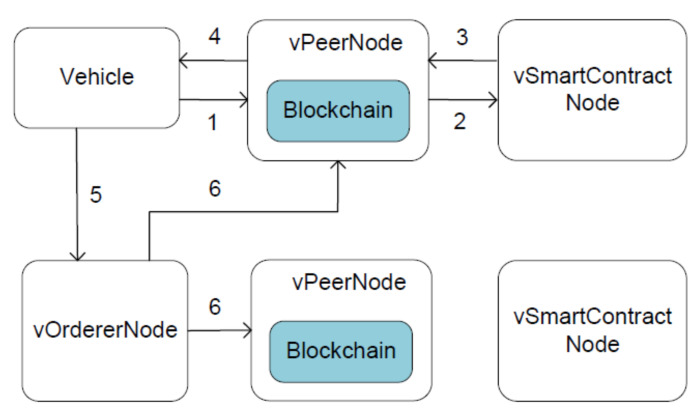
How to disseminate a report via blockchain.

All reports have the same lifecycle [35], which is demonstrated in Figure 7. First, the client application submits a report request to any vPeerNode when a vehicle wants to report a traffic event. Note that this is where the vLoadBalancer comes into play, and any vPeerNode can do the same thing. Second, the vPeerNode checks the validity of the incoming request: format, signature, and access permission. Then the request is formatted as a remote procedure call and is processed by the vSmartContractNode. Third, the vSmartContractNode generates a report response. Fourth, vPeerNode returns the report response to the client. Fifth, the client checks the signature of the incoming response, packages the response and the signature into a report transaction, and sends the transaction to the vOrdererNode. Sixth, the vOrdererNode simply collects transactions from all clients, orders them chronologically, produces blocks of transactions, and delivers these blocks to all vPeerNodes in the network. Finally, every vPeerNode checks the validity of the incoming blocks, including signature and version number, and commits them to the blockchain.

### 5.3. Performance Analysis

The consensus protocol contributes significantly to the performance of our blockchain. PoA is fast and energy efficient because blocks can be generated immediately by designated orderers. By contrast, PoW is slow and energy-intensive because all peers are busy solving the hash problem, and it is accepted by cryptocurrencies like Bitcoin and Ethereum [23,37].

Moreover, two parameters of Hyperledger Fabric affect the performance seriously, i.e., batch timeout and batch size. The former denotes the maximum time to wait before creating a block, and the latter is the maximum number of report transactions into a block. The block is generated whichever is satisfied first. We experiment on the blockchain with the parameters varying, as shown in Section 7.

## 6. Bayesian Network

This section concentrates on how to use Bayesian network in fake news detection. To be specific, we investigate how the submitted reports on the traffic event affect our judgement about the presence of the event. The vSmartContractNode, a component of the blockchain module in an SDRSU, implements the algorithm of Bayesian network, and probability updating is performed whenever a new block is committed.

### 6.1. Network Structure

**Figure 8:**
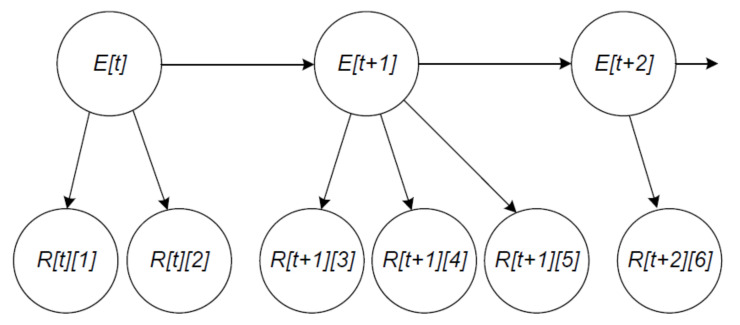
Bayesian network for fake news detection.

While more reports on a traffic event are committed on the blockchain over time, SDRSUs take these reports as evidence and calculate the probability of the presence of the event via our Bayesian network, which is shown in Figure 8.

There are two kinds of nodes in the figure. We establish the hypothesis variables with states present and absent, denoting the traffic states every minute. For example, E[t] denotes the traffic state at time *t*. Note that *t* is the time when the first report on the traffic event is submitted. For demonstration purpose, the Bayesian network in Figure 8 only describes the traffic states in three minutes. We establish the information variables with states positive and negative, denoting the assessments in the gathered reports, whose data structure is illustrated in Figure 6. For instance, R[t][1] denotes the report that is submitted by vehicle $v_{1}$ at time *t*.

There are two types of links in the figure. The links between hypothesis variables and information variables indicate that the state of events has an impact on the state of reports. For example, three reports R[t][1], R[t][2], and R[t][3] are submitted in a minute due to the traffic state E[t]. The links between two hypothesis variables indicate the assumption that the traffic state of the previous minute has influence on the traffic state of the current minute, which in turn has influence on the traffic state of the next minute. That is, our network assumes the Markov property. For instance, the knowledge of E[t] can be used to infer E[t+1].

### 6.2. Conditional Probabilities

To estimate the conditional probabilities or the parameters for Bayesian network in Figure 8, we make three assumptions here. First, the traffic event happens with a Poisson distribution at an average of *n* per month. Second, the traffic event lasts with an exponential distribution at an average duration *t* minutes. Third, the trustworthiness of vehicles is known. By the way, our previous work focuses on deriving the global trust of vehicles [38].

The conditional probability of node E[t] reduces to the prior probability P(E[t]) since it has no parents. An estimate of the prior probability relates to the arrival rate of the traffic event. The mean number of the traffic events that appear in a minute is given by

(9) $$\lambda =\frac{n}{30\times 24\times 60}=\frac{n}{43200}$$

where we assume that there are 30 days in a month. P(E[t]) is equivalent to the probability that at least one event happens in a minute, denoted by

(10) $$P\left(E\left[t\right]\right)=P\left(X\geq 1\right)=1-P\left(X=0\right)=1-e^{-\lambda }$$

where *X* is the number of traffic events in a minute.

We consider P(E[t+1]|E[t]) under two scenarios. When E[t]=present, the conditional probability relates to the duration of the traffic event. The mean number of the traffic events that disappear in a minute is given by

(11) $$\mu =\frac{1}{t}$$

Then P(E[t+1]|E[t]) is equivalent to the probability that the traffic event lasts at least 2 min due to the memoryless property of our network, denoted by

(12) $$\begin{array}{ll} P(E[t+1]|E[t]=present) & =P(T\geq 2)\\ & =\int_{2}^{\infty }\mu e^{-\mu T}dT=e^{-2\mu } \end{array}$$

where *T* is the duration of traffic events. When E[t]=absent, the conditional probability is identical to Equation (10), given by

(13) $$P\left(E\left[t+1\right]|E\left[t\right]=absent\right)=1-e^{-\lambda }$$

Note that Equations (12) and (13) are irrelevant to time *t*. That is, all links between two hypothesis variables have the same strength.

When we compare E[t] with R[t][1], there are four possible results, i.e., true positive, true negative, false positive, and false negative. We use the trustworthiness to reflect the extent to which vehicles correctly report the traffic events. It is drawn from long-term observation and normalized between 0 and 1. For vehicle $v_{1}$, the conditional probabilities can be given by

(14) $$\begin{array}{cc} P(R[t][1]=positive|E[t]=present) & =w_{1} \end{array}$$

(15) $$\begin{array}{cc} P(R[t][1]=negative|E[t]=absent) & =w_{1} \end{array}$$

(16) $$\begin{array}{cc} P(R[t][1]=positive|E[t]=absent) & =1-w_{1} \end{array}$$

(17) $$\begin{array}{cc} P(R[t][1]=negative|E[t]=present) & =1-w_{1} \end{array}$$

where $w_{1}$ is the trustworthiness of $v_{1}$. For other vehicles, we can easily obtain similar formulas.

### 6.3. Probability Updating

This section introduces the basic idea for probability updating. We take the Bayesian network in Figure 8 as an example and calculate the posterior probability P(E[t+2]|x). The evidence *x* is accumulated until time t+2 and given by

(18) $$\begin{array}{ll} x= & \{R[t][1]=r[t][1],R[t][2]=r[t][2],R[t+1][3]=r[t+1][3],R[t+1][4]=r[t+1][4],\\ & R[t+1][5]=r[t+1][5],R[t+2][6]=r[t+2][6]\} \end{array}$$

where r[t][1],r[t][2],r[t+1][3],r[t+1][4],r[t+1][5],r[t+2][6]∈{positive,negative} represent the traffic reports submitted by six vehicles at different time periods.

The unique joint probability distribution P(U) of our network is given by the product of all conditional probabilities specified in Section 6.2. Based on the chain rule for Bayesian network, we have

(19) $$\begin{array}{ll} P(U) & =P(E[t],E[t+1],E[t+2],R[t][1],R[t][2],R[t+1][3],R[t+1][4],R[t+1][5],R[t+2][6])\\ & =P(E[t])P(E[t+1]|E[t])P(E[t+2]|E[t+1])P(R[t][1]|E[t])P(R[t][2]|E[t])P(R[t+1][3]|E[t+1])\\ & \;\;P(R[t+1][4]|E[t+1])P(R[t+1][5]|E[t+1])P(R[t+2][6]|E[t+2]) \end{array}$$

After inserting the evidence *x*, we obtain

(20) $$\begin{array}{ll} P(U,x) & =P(E[t],E[t+1],E[t+2],r[t][1],r[t][2],r[t+1][3],r[t+1][4],r[t+1][5],r[t+2][6])\\ & =P(E[t])P(E[t+1]|E[t])P(E[t+2]|E[t+1])P(r[t][1]|E[t])P(r[t][2]|E[t])P(r[t+1][3]|E[t+1])\\ & \;\;P(r[t+1][4]|E[t+1])P(r[t+1][5]|E[t+1])P(r[t+2][6]|E[t+2]) \end{array}$$

According to Bayes’ theorem, P(E[t+2]|x) is calculated via

(21) $$\begin{array}{ll} P(E[t+2]|x) & =\frac{P(E[t+2],x)}{P(x)}\\ & =\frac{\sum_{\left\{E[t],E[t+1]\right\}}P\left(U,x\right)}{\sum_{\left\{E[t],E[t+1],E[t+2]\right\}}P\left(U,x\right)} \end{array}$$

where P(E[t+2],x) and P(x) are derived from P(U,x) via marginalization [39].

## 7. Simulations and Experiments

This section evaluates the performance of QcFND from three aspects, waiting time, transaction time, and accuracy. The waiting time refers to the time between when traffic reports arrive at the ports of SDRSUs and when they can be actually processed by blockchain nodes. The transaction time refers the time we need to write the reports on the blockchain. The waiting time and transaction time constitute the main part of response time $T_{r}^{tra}$ or $T_{r}^{sdn}$. The accuracy relates to how well the truth can be discovered from the received reports.

We utilize three experimental platforms to carry out the evaluations respectively, i.e., Veins [40], Hyperledger Fabric [35], and Netica [41]. Veins is an open-source framework for running vehicular network simulations, and it is based on two simulators: OMNeT++ [42] and SUMO [43]. Hyperledger Fabric is an enterprise-grade permissioned distributed ledger platform. Netica is a comprehensive program for working with Bayesian networks.

**Figure 9:**
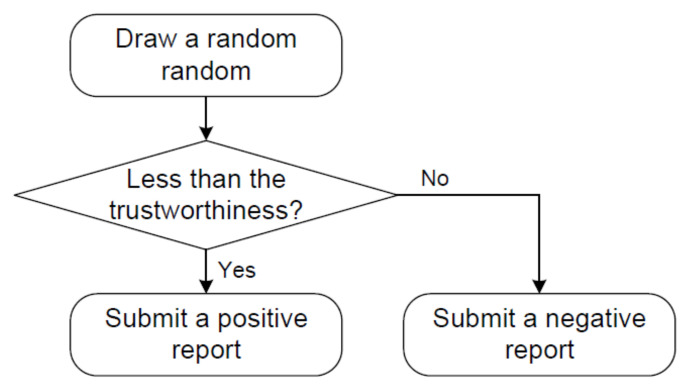
How to simulate traffic reports.

Figure 9 illustrates how to generate the simulated reports when we assume that a traffic event happens. For each vehicle, we draw a random number from [0,1], which is compared with the trustworthiness of the vehicles. If the random number is less than the trustworthiness, the vehicle submits a positive report; otherwise, it submits a negative report. For instance, it is likely that fake news is submitted when the trustworthiness is low. While the three experimental platforms experiment with these reports, the performance of QcFND is tested and the results are analyzed.

### 7.1. Waiting Time

The waiting time is measured under two scenarios, with and without the load balancer. We implement the load balancing of SDN network in OMNeT++.

#### 7.1.1. Traffic Network

**Figure 10:**
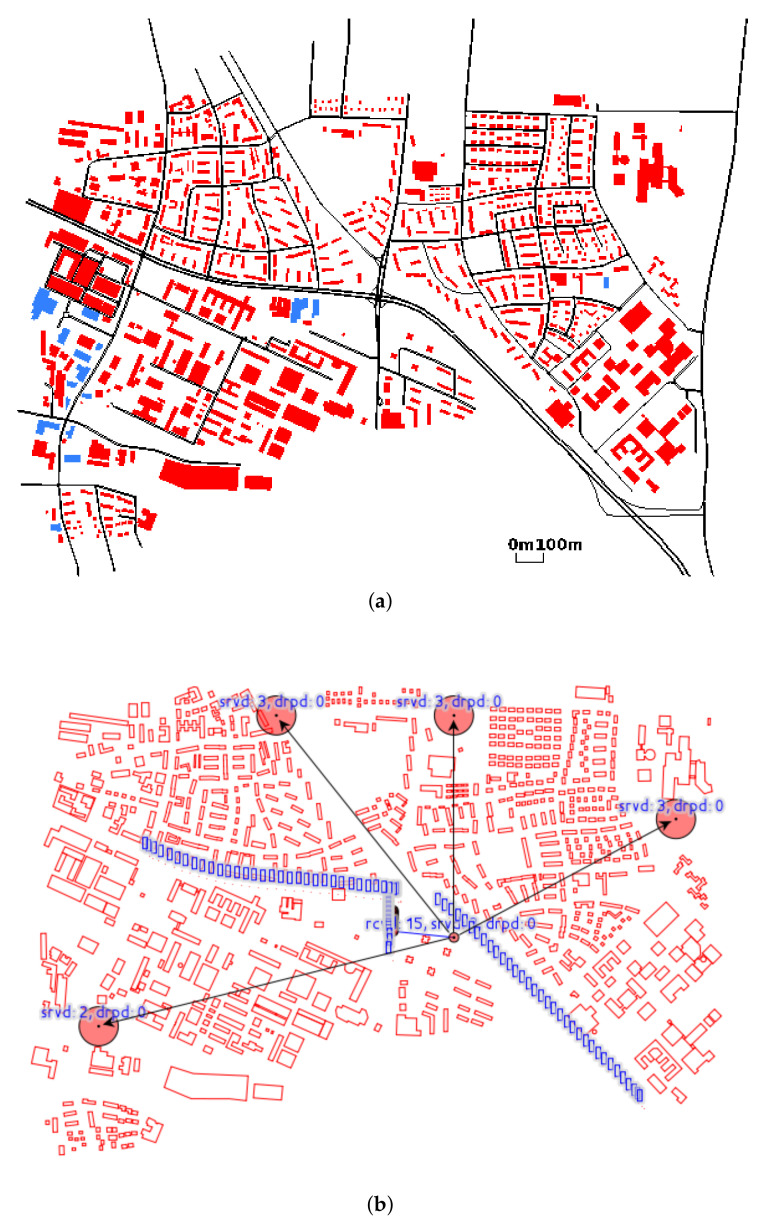
The load balancing implemented in IoV. (**a**) Traffic network in Erlangen. (**b**) SDN network.

We simulate road traffic using a real map of the German city of Erlangen, which is shipped with Veins and shown in Figure 10a. The study area is about 2000 m long and 2700 m wide. It consists of a set of urban roads, intersections and obstacles. There is an accident near the crossroad at the center of the figure. Red polygons indicate buildings, which are obstacles in wireless communication. The traffic demand consists of 250 vehicles, each of which is going to pass the accident spot. Once they observe the accident, they submit the reports on the accident to the nearest SDRSU.

#### 7.1.2. Communication Network

Vehicles and SDRSUs implement the Intelligent Transport System (ITS) protocol stack, which uses IEEE 802.11p and IEEE 1609.4. Vehicles are represented by small blue boxes in Figure 10b. There are five SDRSUs in the figure, represented by red circles. The SDRSU denoted by the small red circle is connected with other four SDRSUs denoted by the big red circles, and there is a 0.1 s delay on the connections. The communications between vehicles and SDRSUs are denoted by blue lines in Figure 10b. We adopt default configurations in Veins. The path loss and shadowing models are enabled, as they have influence on the wireless communication.

SDRSUs implement the functionality of load balancing, as described in Section 4. Due to the accident, vehicles may generate more reports than an SDRSU can handle. Hence, a simple load balancing policy is enforced. We choose the SDRSU with the shortest waiting queue when forwarding the incoming reports. Note that the maximum size of the buffer queue in SDRSU is set to 10.

#### 7.1.3. Results

Table 4 demonstrates the simulation results, which are obtained in the duration of each simulation cycle, 240 s. Two settings have a significant impact on the waiting time and the report loss, i.e., processing time and load balancing. The former ranges from 0.5 s to 3 s, and the latter indicates whether the load balancer is enabled. This paper tests the performance of network with these parameters varying.

The first row indicates the case where it takes 0.5 s to process a report and the load balancer is not implemented. There are 166 reports submitted by vehicles, and the mean arrival time of these reports is at t=148.355 s. There are 166 reports served by traditional RSUs, and the mean leave time of these reports is at t=148.953 s. Therefore, we obtain the waiting time 0.098 s, which is calculated via 148.953−148.355−0.5. In addition, the number of dropped reports is 0. In the second row, the load balancer is implemented in SDRSUs and the mean leave time is at t=148.934 s.

In the third row, there are 164 served reports and the mean leave time of these reports is at t=153.110 s. Since there are two dropped reports due to the overloads, the mean waiting time of 166 reports is unavailable. In contrast, there is no dropped report due to the load balancer, and the waiting time is 0.079 s.

We can see that the number of dropped reports increases with the increase of processing time when the load balancer is not enabled. However, when the load balancer is enabled, there is no dropped report and the waiting time increases a little with the increase of processing time. In conclusion, the performance of IoV is greatly improved because of the introduction of SDRSUs.

### 7.2. Transaction Time

The transaction time is measured with two parameters varying. The blockchain adopts PoA as its consensus mechanism to quickly share the submitted reports across IoV.

#### 7.2.1. Experimental Environment

Our blockchain is deployed on a machine with Intel Xeon E5-26xx v4 2.4 GHz CPU and 2 GB RAM running Ubuntu 16.04.1 LTS. All virtual servers are built with Docker 18.06.1-ce, that is, they are virtualized into containers sharing the hardware and the operating system kernel [44]. We create five vPeerNodes and one vOrdererNode. A certificate authority (CA) dispenses X.509 certificates used to identify servers and clients. X.509 certificates are used in the lifecycle of transactions. The blockchain network is created by Docker Compose, which is a tool for defining and running multi-container Docker applications.

#### 7.2.2. Results

Table 5 demonstrates the transaction time in three cases. The results are averages and obtained through running the write transactions three times. Note that CA–CE indicate the commitments on five vPeerNodes.

The table shows the timing of write transactions under the condition of the parameters batch size and batch timeout, including the time of endorsement on an endorsing peer, block generation on the orderer, and commitment on all peers. It can be seen that the transaction time depends on the two parameters. For example, the first row shows the process as follows. A vPeerNode accepts a report on a traffic event at t=0 s, and it then endorses the report. The vOrdererNode generates a block containing the report transaction at t=20.042 s. Subsequently, five vPeerNodes commit the block to the blockchain at t=20.230,20.206,20.246,20.241,20.220 s, respectively. Therefore, the report transaction is available on all peers at the latest time, namely t=20.246 s. Consequently, the transaction time of 20.246 s is obtained with batch timeout equal to 20 s and batch size equal to 1000. The second and third rows are with the transaction time 0.290 and 2.194 s, respectively.

We can explain the transaction latency in Table 5. In the first and third row, the vOrdererNode has to wait 20 s and 2 s respectively before creating a block because there is only one incoming report transaction that needs to be packaged into the block and the batch timeout occurs first. In the second row, the batch size is 1 and there happens to be one report transaction, so the batch size is satisfied first and the vOrdererNode does not have to wait 20 s before creating a block. In conclusion, we can configure the batch size and the batch timeout to adjust the transaction time as acquired.

#### 7.2.3. Comparison to Existent Works

The consensus mechanisms adopted by different blockchain networks contribute significantly to the performance difference. QcFND chooses PoA, which is fast and energy efficient because blocks can be generated immediately by designated orderers. Bitcoin and Ethereum adopt PoW, which is slow and energy-intensive because all peers are busy solving the hash problem. Bitcoin and Ethereum have to take 600 s and 10 s respectively to write a transaction on the blockchain [27]. The scheme from [24] uses PoW as well, and its write latency is 600 s. The scheme of [31] adopts PoE, and it takes 22.4 s for 16 events in an hour to be synchronized across 160 nodes. A joint PoW and PoS is used in [32], resulting in the adaptive block generation time.

### 7.3. Accuracy

In Section 6, we calculate the posterior probability of traffic events given submitted reports. Actually, the accuracy and the posterior probability are equal in amount as they both denote the coherence between the truth and the guess. This section investigates how the accuracy changes with the accumulation of evidence.

#### 7.3.1. Simulation Settings

**Figure 11:**
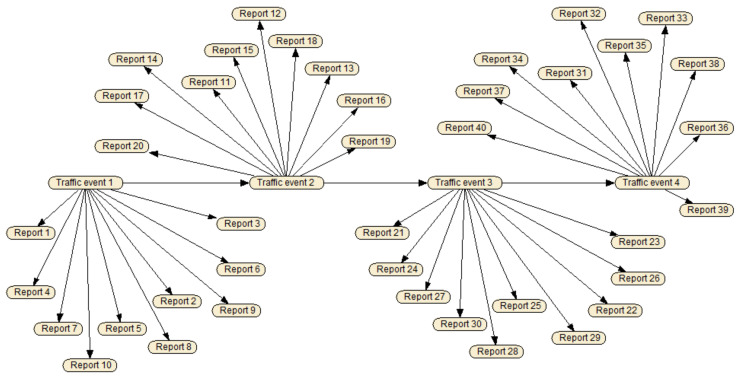
Screenshot of Bayesian network for QcFND.

Figure 11 demonstrates the Bayesian network that we create with Netica. Traffic events 1–4 represent the traffic state in four consecutive minutes; reports 1–40 denote the traffic reports submitted by 40 vehicles with different trust levels in the same time period.

It is assumed that a kind of traffic event happens 10 times a month and the average duration of the traffic event is 20 min. According to Equations (9) and (11), we have λ=0.000231 and μ=0.05. Therefore, the probability that the traffic event happens in a minute is P(E[t])=0.000231 via Equation (10), and the probability that the traffic event will persist for the next minute is P(E[t+1]|E[t])=0.904837 via Equation (12). The trustworthiness of vehicles is drawn from a truncated normal distribution. The mean of the distribution ranges from 0.1 to 0.9, in step of 0.1, the standard deviation is set to 0.1 and the domain is restricted to [0,1]. Hence, we get the conditional probability in Equations (14)–(17). It is also assumed that there are 10 reports gathered in a minute.

**Figure 12:**
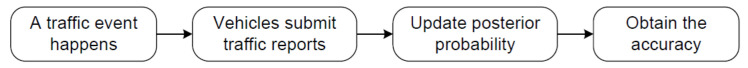
How to obtain the accuracy.

Figure 12 illustrates how to obtain the accuracy. First, we assume a traffic event happens. Second, vehicles submit reports on the event based on their trust levels. Third, the posterior probability is updated via Bayesian network whenever a report is received. Fourth, the derived posterior probability is taken as the accuracy since it estimates the probability of a traffic event that is assumed to be present.

#### 7.3.2. Results

**Figure 13:**
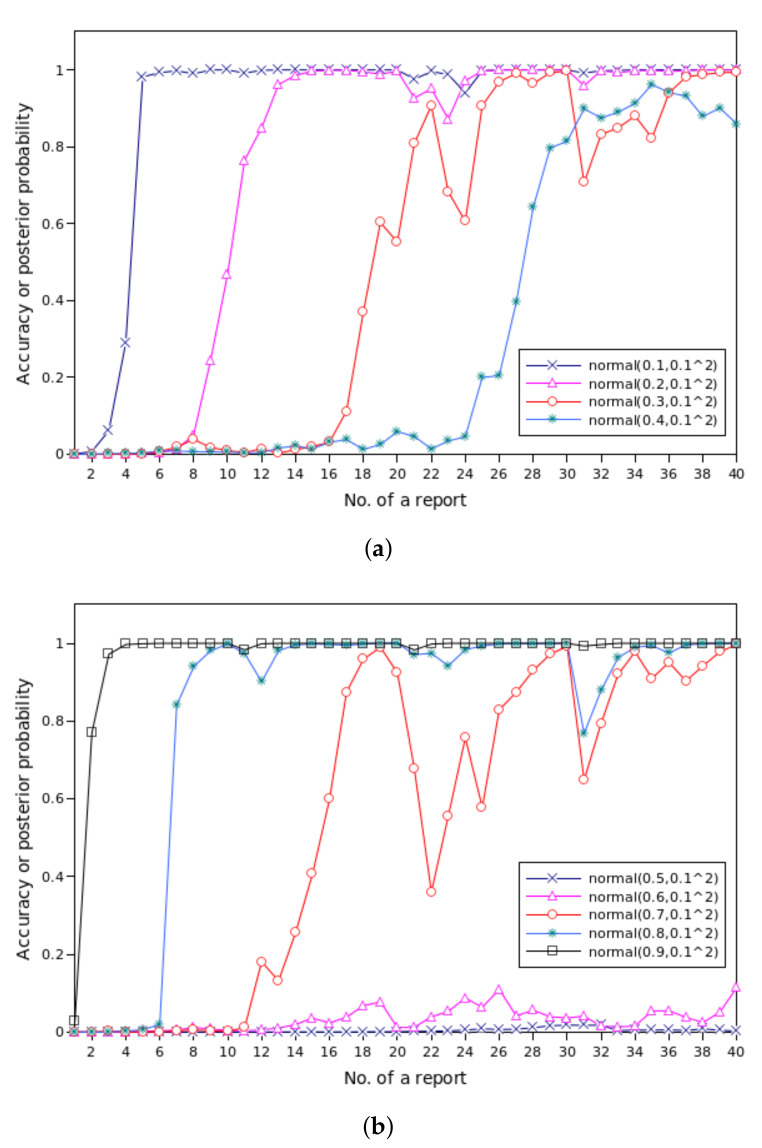
The accuracy or posterior probability of QcFND. (**a**) Vehicles with low level of trust. (**b**) Vehicles with high or middle level of trust.

The results are shown in Figure 13. In each simulation cycle, there is a total of 40 reports received in four minutes. We experiment with the population of vehicles whose trustworthiness is drawn from different normal distributions, and the posterior probability may rise and fall over time as more and more reports are collected.

In Figure 13a, it can be observed that QcFND reaches high level of accuracy even if vehicles are with low levels of trust. When the trustworthiness is drawn from $normal(0.1,0.1^{2})$, we get the results close to 1 since the 5th report, which accurately represents the truth. That is, QcFND gives the correct results quickly. Most results are greater than 0.9 since t=2 min when the trust levels obey $normal(0.2,0.1^{2})$, which is good enough to reflect the truth. In other words, we achieve the correct results one minutes after the presence of the traffic event.

When the trustworthiness comes from $normal(0.3,0.1^{2})$ or $normal(0.4,0.1^{2})$, most results at t=4 min are greater than 0.8, which is good enough to reflect the truth. That is, we achieve the satisfactory accuracy four minutes after the traffic event happens, although there are some vibrations beforehand.

In Figure 13b, we can see that QcFND reaches high level of accuracy when vehicles are with high levels of trust. When the trustworthiness is drawn from $normal(0.9,0.1^{2})$, we get results close to 1 from the 3rd report, which accurately represents the truth. That is, the system almost gives the correct results immediately. Most results are greater than 0.9 since t=2 min when the trust levels obey $normal(0.8,0.1^{2})$, which is good enough to reflect the truth. In other words, we achieve the correct results one minute after the presence of the traffic event.

When the trustworthiness comes from $normal(0.7,0.1^{2})$, most results at t=4 min are greater than 0.9, which is good enough to reflect the truth. That is, we achieve the satisfactory accuracy four minutes after the traffic event happens, although there are sharp vibrations beforehand. When the trustworthiness obeys a normal distribution $normal(0.5,0.1^{2})$ or $normal(0.6,0.1^{2})$, the accuracy remains very low. The results cannot reflect the truth at all since it says the probability of the traffic event is negligible. In other words, vehicles with the mean 0.5 or 0.6 cannot provide useful information to support the estimation of traffic events.

In conclusion, the trustworthiness of vehicles has a significant impact on the timeliness and accuracy of QcFND in Bayesian network. If the trust levels are far away from the intermediate value 0.5, QcFND needs less time and less evidence to find the truth.

#### 7.3.3. Comparison to Existent Works

Here we compare the performance of QcFND with a few works introduced in Section 2.4 based on the experimental results.

In [4], the minimum counting threshold is 5, which means that at least 5 traffic reports from the vehicles that detect the warning accident are needed to evaluate the credibility of the warning event. In contrast, QcFND only needs 3 traffic reports to estimate the probability of the warning event if these reports are from vehicles with high trust levels. Therefore, QcFND can obtain accurate results via less evidence, resulting in faster response time.

In [5], the decision accuracy decreases with the number of vehicles with low trust levels, which give wrong opinions on the traffic accident. However, it is not the case for QcFND since we can still obtain accurate results even if traffic reports are from untrustworthy vehicles, as shown in Figure 13a. For instance, if a vehicle with a low trust level submits a negative traffic report, it is reasonable to draw the conclusion that the traffic accident has very likely happened.

In [29], the message detection accuracy never reaches 1, which means there is always an opportunity that we make wrong judgement about traffic events. Besides, the accuracy drops rapidly with the growing capacity of untrusted vehicles. In contrast, QcFND achieves totally correct results when the reports are from vehicles with high or low trust levels.

In [31], the event success rate is affected seriously by the threshold of the 2nd pass, which is not easy to configure. Moreover, the accuracy also drops dramatically when the percentage of untrusted vehicles increases. By contrast, QcFND has no parameters that need to be preset based on trial and error, and obtains accurate results even when untrusted vehicles are present.

## 8. Conclusions

This paper aimed to address the problem of how to quickly detect the fake news in IoV. Hence, we proposed a network computing framework QcFND to achieve the goal, which utilizes the technologies from SDN, edge computing, blockchain, and Bayesian networks. First, we designed an SDIoV, which is composed of interconnected SDRSUs and implements the load balancing to speed up the processing of reports. Second, we designed a blockchain, which was deployed on the SDRSUs and adopts PoA as its consensus mechanism to share the reports quickly and securely. Third, the algorithm of Bayesian network was also deployed on the SDRSUs, which calculates the posterior probability of a traffic event considering the prior probability and the duration of the traffic event, the trustworthiness of vehicles, and the collected reports. The simulation results demonstrate the improvement in the performance of QcFND from three aspects, i.e., waiting time, transaction time, and accuracy.

Despite the work done in this paper, there is still further research ahead. First, a complicated load balance algorithm needs to be considered. For example, the geographical distance between SDRSUs can be taken into consideration. Second, we need a method to determine which SDRSU hosts the vOrdererNode. Third, we can explore more appropriate ways to acquire the parameters of our Bayesian network. Fourth, QcFND needs to be tested based on real-world cases to further validate its effectiveness. Fifth, we can deploy QcFND in a production environment to further investigate its performance.

## Figures and Tables

**Table 1:** Comparison of the proposed trust model with the existing schemes.

Reference	Method Used	Defense against Sybil Attack	Basis for Trust Decision
[28]	Dempster–Shafer theory	Partly	Data sensed and collected by vehicles
[2]	Empirical formula	Partly	Trustworthiness of vehicles, relation between events and vehicles,
			and proximity in location and time
[29]	Reputation-determined	Yes	Observation of traffic environment
[5]	Weighted voting	Yes	Observation of traffic environment
[30]	Majority voting	Yes	Observation of traffic environment
[31]	Proof-of-Event	Yes	Threshold-based event validation algorithm
[4]	Counting	Yes	Observation of traffic environment
[32]	Bayesian inference	Partly	Observation of traffic environment and proximity in location
Proposed scheme	Bayesian network	Yes	Trustworthiness of vehicles, characteristics of events,
			and causal relationship among events, vehicles, and reports

**Table 2:** Extended MAC table.

MAC Address	Ingress Port	Queue Size
12-34-56-78-90-AB	b	3
12-34-56-78-90-AC	c	1
12-34-56-78-90-AD	d	2

**Table 3:** Messages used by communication protocol.

Message Name	Direction	Description
SDNP_Queue_Size	switch-to-controller	The SDN switch broadcasts the size of its waiting queue to the network whenever the size changes.
SDNP_Packet_In	switch-to-controller	The SDN switch asks the vSDNController how to deal with its just received message.
SDNP_Balance_In	controller-to-balancer	The vSDNController asks the vLoadBalancer how to deal with the message considering load balancing.
SDNP_Balance_Out	balancer-to-controller	The vLoadBalancer tells the vSDNController to forward the message
		via a specified port considering load balancing.
SDNP_Packet_Out	controller-to-switch	The vSDNController tells the SDN switch to forward the message via a specified port.

**Table 4:** The waiting time and the report loss.

Processing Time (s)	Load Balancing	Received (#)	Arrival Time (s)	Served (#)	Leave Time (s)	Waiting Time (s)	Dropped (#)
0.5	No	166	148.355	166	148.953	0.098	0
0.5	Yes	166	148.355	166	148.934	0.079	0
1	No	166	148.355	164	153.110	n/a	2
1	Yes	166	148.355	166	149.434	0.079	0
2	No	166	148.355	96	143.956	n/a	61
2	Yes	166	148.355	166	150.434	0.079	0
3	No	166	148.355	66	142.704	n/a	91
3	Yes	166	148.355	166	151.439	0.084	0

**Table 5:** The transaction time of a report.

Batch Size (#)	Batch Timeout (s)	Endorsement (s)	Ordering (s)	CA (s)	CB (s)	CC (s)	CD (s)	CE (s)	Transaction Time (s)
1000	20	0	20.042	20.230	20.206	20.246	20.241	20.220	20.246
1	20	0	0.048	0.290	0.278	0.273	0.282	0.289	0.290
10	2	0	2.038	2.184	2.191	2.180	2.193	2.194	2.194
